# Oral Health Status and Factors Related to Oral Health in Patients with Schizophrenia: A Matched Case-Control Observational Study

**DOI:** 10.3390/jcm13061584

**Published:** 2024-03-10

**Authors:** Reza Aghasizadeh Sherbaf, George Michael Kaposvári, Katalin Nagy, Zoltán Péter Álmos, Zoltán Baráth, Danica Matusovits

**Affiliations:** 1 Department of Oral Surgery, Faculty of Dentistry, University of Szeged, Tisza Lajos krt. 64–66., 6720 Szeged, Hungary; aghasizadeh.reza@stoma.szote.u-szeged.hu (R.A.S.); kaposvari.george@stoma.szote.u-szeged.hu (G.M.K.); katalin.nagy@universityszeged.com (K.N.); 2 Department of Psychiatry, Faculty of Medicine, University of Szeged, Korányi fasor 8–10., 6720 Szeged, Hungary; almos.peter@med.u-szeged.hu; 3 Department of Prosthodontics, Faculty of Dentistry, University of Szeged, Tisza Lajos krt. 64–66., 6720 Szeged, Hungary; barath.zoltan@stoma.szote.u-szeged.hu

**Keywords:** schizophrenia, mental disorder, oral health, dental status, periodontal status, DMF-T, case-control study

## Abstract

**Background:** Schizophrenia (SCZ) patients have disproportionately poor oral health outcomes owing to a multidimensional set of factors, such as pathophysiology of the disease, drug-related adverse effects and lower utilization rate of dental healthcare services. The aim of the present observational study was to compare the indicators of dental and periodontal health in patients with SCZ to those of nonaffected healthy controls; furthermore, the influence of various anamnestic factors and lifestyle habits on oral health status were also assessed. **Methods:** A total of 50 SCZ patients—in remission—receiving treatment at the Department of Psychiatry, University of Szeged, were compared with 50 age- and gender-matched healthy controls attending the Faculty of Dentistry, University of Szeged. Participants’ dental (decayed, missing and filled surfaces [DMF-S] and decayed, missing and filled teeth [DMF-T]) and periodontal (plaque index [%], bleeding on probing [BOP%], pocket depth [PD] and attachment loss [AL]) status was measured according to the World Health Organization (WHO) criteria. **Results:** In total, 74.0%, 80.0% and 78.0% of SCZ patients received second-generation antipsychotics, benzodiazepines and mood stabilizers, respectively. Patients with SCZ had significantly higher DMFs (81.30 ± 40.16 vs. 61.64 ± 40.56; *p* = 0.010), D (8.18 ± 7.73 vs. 4.18 ± 4.22; *p* < 0.001) and DMF-T (18.20 ± 8.36 vs. 14.42 ± 8.21; *p* = 0.024) scores but significantly lower F (1.84 ± 0.29 vs. 4.62 ± 3.98; *p* < 0.001) scores compared to the controls; male subjects had significantly lower DMFs (74.52 ± 39.72 vs. 90.67 ± 39.1; *p* = 0.020) and DMF-T (16.52 ± 8.12 vs. 20.52 ± 8.32; *p* = 0.031) scores. Additionally, SCZ patients had significantly higher plaque indices (56.96 ± 23.19 vs. 27.44 ± 17.53; *p* < 0.001), BOP% (58.96 ± 22.89 vs. 23.56 ± 17.53; *p* < 0.001), PD (2.84 ± 0.67 vs. 2.19 ± 0.49; *p* = 0.024) and AL (3.39 ± 1.72 vs. 2.49 ± 0.76; *p* < 0.001) values compared to controls. Smoking > 10 cigarettes/day was associated with worse dental and periodontal indices, while consuming ≥ 4 units/week of alcohol was associated with worse periodontal indices, respectively (*p* < 0.05 in all cases). In contrast, coffee consumption rates and vitamin supplementation status had no significant effect on oral health status indicators. **Conclusions:** Our study highlights the overall poor oral health status of individuals affected by SCZ and the need for targeted preventive interventions.

## 1. Introduction

Mental disorders constitute a considerable and increasing burden on global health, both from an epidemiological and economic context [1]; according to the Global Burden of Disease Study (GBD) 2019 estimates, they account for over >400 million disability-adjusted life years (DALYs; 16% of the global burden), and ~USD 5 trillion of overall economic costs [2]. Mental disorders include depressive, bipolar and anxiety disorders; substance abuse disorders; eating disorders; schizophrenia (SCZ); and self-harm, among others, all of which are often associated with poor quality of life (QoL) [3,4]. SCZ is a complex mental disorder—which affects 0.45% (1:222) of the adult population—characterized, on one hand, by persistent hallucinations, delusions, psychosis, disorganized behavior and agitation (i.e., positive symptoms) and, on the other hand, by social withdrawal, limited experience of emotions, passiveness, cognitive dysfunctions and slow movements (i.e., negative symptoms) [5,6]. SCZ patients are often affected by discrimination and stigma, further facilitating withdrawal from society and from performing everyday tasks [7]. Based on a recent meta-analysis, 24.2% (95% CI: 20.3–28.0%) of SCZ patients returned to previous functionality following treatment [8]; however, it has been estimated that the risk of relapse in SCZ is ~3.5%/month [9].

Many individuals affected by SCZ also suffer from various comorbid conditions, including cardiovascular diseases, chronic obstructive pulmonary disease (COPD), obesity, diabetes, endocrine disorders and other mental health conditions (e.g., mood disorders, panic disorders, substance abuse) [10,11]. Additionally, many reports highlight the disproportionately poor oral health outcomes (including higher incidence and severity of dental caries, periodontal disease and severe tooth loss) of SCZ patients, owing to a multidimensional set of factors [12,13]. Oral health is a critical aspect of an individual’s overall health and QoL, having essential physiological (breathing, eating), social (communication) and psycho-social (confidence, well-being) dimensions [14,15]. Furthermore, the bidirectional relationship between mental health indicators and overall dental health status has also been noted previously [16,17]. A variety of factors may contribute to the poor oral health of SCZ patients: they may lack motivation to keep up adequate oral hygiene habits (due to negative symptoms or a relapse) [18], and they are often less likely to attend dental visits than their healthy counterparts [19]. SCZ patients often receive medications, including first- and second-generation antipsychotics, mood stabilizers, benzodiazepines and anti-Parkinson drugs (e.g., anticholinergics), all of which present with numerous adverse effects in the oral cavity [20]. Salivary gland hypofunction and xerostomia are notable consequences of these drugs, leading to increased incidence of (often severe) caries due to the reduced salivary flow rate [21]. Likewise, orofacial (or tardive) dyskinesia constitutes involuntary movements of the face, mouth and tongue, which hinders the individual’s ability to perform oral hygiene practices effectively [22]. On the other hand, various lifestyle factors, such as tobacco consumption, alcohol use and poor dietary habits (i.e., high intake of simple sugars, low intake of fibers and vitamins), may further exacerbate the oral health (high rates of caries, poor periodontal status, experience of ulcers) of SCZ patients [23,24,25].

As individuals affected by mental disorders often receive suboptimal dental healthcare—especially in the case of hospitalized patients—many initiatives have been put forth aiming to recognize and highlight the vulnerability of specific psychiatric patient groups—including SCZ patients—to dental caries, deterioration in periodontal status, tooth loss and poor QoL [26]. As such, these patients may require a heightened focus on preventive oral hygiene interventions, or dental treatment for oral health rehabilitation. While there has been an increase in the number of clinical studies dealing with the oral health status of patients with SCZ [27,28], there are limited epidemiological data available comparing the dental and periodontal parameters of patients with SCZ to the general population. Therefore, the aim of the present observational study was to compare the indicators of dental and periodontal health in patients with SCZ to those of nonaffected healthy controls. In addition, the influence of various anamnestic factors and lifestyle habits on oral health status were also assessed. Our working hypotheses were as follows: (*i*) SCZ patients have worse dental status parameters compared to their healthy controls, (*ii*) SCZ patients have worse periodontal status parameters compared to their healthy controls, (*iii*) lifestyle habits may have negative (i.e., tobacco consumption, alcohol consumption, coffee consumption) or positive (i.e., use of vitamin supplements) effects on dental and/or periodontal status.

## 2. Materials and Methods

### 2.1. Study Design and Inclusion Criteria

The present single-center case-control observational study was carried out at the Albert Szent-Györgyi Health Centre, University of Szeged, a primary- and tertiary-care teaching hospital situated in the Southern Great Plain of Hungary. Study participants comprised two cohorts: (*i*) Cases, i.e., individuals diagnosed with SCZ, treated as outpatients or inpatients at the Department of Psychiatry, University of Szeged, Hungary. The diagnosis of SCZ was based on the presence of positive symptoms (e.g., hallucinations and delusions), negative symptoms (e.g., diminished emotional expressivity and avolition) and social/occupational dysfunction over a period of time, as specified by the *Diagnostic and Statistical Manual of Mental Disorders* (DSM) of the American Psychiatric Association (APA) [29]. Patients diagnosed with SCZ were selected through a random stratified sampling method—based on age and gender—from a larger pool of patients at the Schizophrenia Ward or the Rehabilitation Center at the Department of Psychiatry [30]. To establish a consistent baseline and to mitigate variations associated with the acute phase of the disease, only SCZ patients in the remission state were eligible to participate [31]. (*ii*) Controls, i.e., generally healthy individuals willing to participate, without a psychiatric disorder (and not taking any psychiatric medications) or other severe systemic illnesses, seeking dental treatment at the Faculty of Dentistry, University of Szeged, Hungary. To ensure the reliability of comparative analyses, individuals in the control groups were matched to SCZ patients according to their age (±2 years) and gender distributions [32]. A required sample size of *n* = 50 participants per study group was determined to ensure that changes of a magnitude of 1.5 in a quantitative variable (e.g., decayed, missing and filled surfaces [DMF-S] and decayed, missing and filled teeth [DMF-T]) would be identified, with a statistical power of 80% and the α set at 5%, as described previously [33]. This study was carried out between 1st of November 2016 and 31st of December 2021.

### 2.2. Assessment of Oral Health Status; Anamnestic Data

The assessment of oral health in SCZ patients and controls was carried out through a comprehensive full-mouth dental status and periodontal examination, according to the World Health Organization (WHO) criteria; during the examination, the following indices were calculated: (*i*) decayed, missing and filled surfaces (DMF-S); (*ii*) decayed, missing and filled teeth (DMF-T); (*iii*) missing teeth (Mt; count of teeth missing); (*iv*) plaque index (%); (*v*) bleeding on probing (BOP%); (*vi*) mean pocket depth (mean PD); (*vii*) mean attachment loss (mean AL); (*viii*) severe tooth loss (having ≤8 remaining teeth); and (*ix*) number of crowns (i.e., count of individual crowns, including both pontic crowns and abutment crowns) [34,35]. Edentulous individuals and their controls were excluded from the analysis of periodontal status parameters.

The clinical oral examinations were conducted in a separate oral and maxillofacial department beside the Department of Psychiatry, University of Szeged. The oral health status assessment of SCZ patients was carried out under the supervision of a psychiatric care physician. The oral cavity was illuminated with a penlight and evaluated visually using a dental mirror, tweezers and a periodontal probe. The clinical examination included using periodontal probes and panoramic radiographs (OPGs). To ensure uniformity, all dental examinations were performed by two operators (R.A.S. and G.M.K.), both with over five years of clinical experience at the time of this study. After the oral examination of the subjects, instructions on maintaining oral hygiene were given by the operators.

In addition to the full oral workup of the SCZ patients and controls, data on anamnestic information (if relevant) and lifestyle habits were also collected during this study, corresponding to factors that could impact oral health outcomes. The following data were collected through direct questions from the examiners: (*i*) gender, (*ii*) age, (*iii*) tobacco consumption (expressed as cigarettes/day), (*iv*) alcohol consumption (expressed as units/week), (*v*) coffee consumption (expressed as cups of coffee/day), (*vi*) use of vitamin supplements (yes/no), (*vii*) use of second-generation antipsychotic medications (e.g., olanzapine, risperidone), (*viii*) use of benzodiazepines (e.g., clonazepam, alprazolam), and (*ix*) use of mood stabilizer medications (e.g., carbamazepine, sodium valproate).

### 2.3. Statistical Analysis

During analysis, all continuous variables were expressed as means and standard deviations (mean ± SD), whereas categorical variables were expressed as frequencies (*n*) and percentages (%). Normality of variables was tested using the graphical method (Q-Q diagrams) and Shapiro–Wilk tests. The Fisher’s exact test was used to detect differences between proportions (with a Cramér’s phi [φ] effect size measure), while Mann–Whitney U-tests were carried out for the comparison of continuous variables between groups of interest. Statistical analyses were performed using SPSS Statistics version 26.0 (IBM Inc., Chicago, IL, USA). During analyses, *p* values < 0.05 were considered statistically significant.

### 2.4. Ethical Considerations

This study was conducted in accordance with the Declaration of Helsinki and national and institutional ethical standards. Ethical approval for the study protocol was obtained from the Human Institutional and Regional Biomedical Research Ethics Committee, University of Szeged, Hungary (reference number: 170/2016-SZTE [3867]), and the Hungarian Medical Research Council (ETT-TUKEB; reference number: IV/2426-2/2020/EKU). Written informed consent was obtained from all the participants involved in this study or their legal guardians. During data collection, the anonymity of the patients and controls was preserved.

## 3. Results

### 3.1. Demographic Characteristics and Anamestic Data

Fifty (*n* = 50) patients with SCZ were included in this study, along with fifty (*n* = 50) matched, healthy control subjects; demographic characteristics were the following for both groups: (*i*) Gender: 29 males [58.0%] and 21 females [42.0%]. (*ii*) Age: the mean age was 51.86 ± 13.28 years (range: 29–80), with the following distribution: <40 years: *n* = 9 (18.0%), 41–59 years *n* = 26 (52.0%), ≥60 years *n* = 15 (30.0%) (no significant differences were present between the two groups on the basis of age [*p* = 1.000] or gender [*p* = 1.000]). A summary of anamnestic and lifestyle characteristics corresponding to SCZ patients and controls is shown in Table 1; significant differences were observed in the context of tobacco consumption (*p* < 0.001, φ = 0.529), alcohol consumption (*p* = 0.038, φ = 0.261), coffee consumption (*p* = 0.004, φ = 0.358) and the use of vitamin supplements (*p* < 0.001, φ = 0.408), respectively. A total of 18.0% and 56.0% of SCZ patients and controls were nonsmokers, respectively. Among the SCZ patients, *n* = 37 (74.0%) patients received second-generation antipsychotic drugs, *n* = 40 (80.0%) received benzodiazepines, while *n* = 39 (78.0%) received mood stabilizers at the time of this study. During comparative analyses, the following subgroups were made: smoking: 0–10 cigarettes/day vs. >10 cigarettes/day, alcohol consumption: 0–3 units/week vs. ≥4 units/week, coffee consumption: 0–1 cups/day vs. ≥2 cups/day, vitamin supplements: taking vs. not taking them.

### 3.2. Dental Status Parameters

The summary of dental status findings corresponding to SCZ patients and controls is presented in Table 2. Patients with SCZ had significantly higher DMF-S (81.30 ± 40.16 vs. 61.64 ± 40.56; *p* = 0.010), D (8.18 ± 7.73 vs. 4.18 ± 4.22; *p* < 0.001) and DMF-T (18.20 ± 8.36 vs. 14.42 ± 8.21; *p* = 0.024) scores but significantly lower F (1.84 ± 0.29 vs. 4.62 ± 3.98; *p* < 0.001) scores compared to the controls. Only numerical differences were shown for the number of crowns (1.76 ± 4.01 vs. 2.88 ± 4.56; *p* = 0.059) and M scores (8.18 ± 7.73 vs. 5.62 ± 6.61; *p* = 0.071), respectively (Table 2). *n* = 42 (84.0%) of SCZ patients and *n* = 36 (72.0%) of controls had at least one missing tooth, while *n* = 26 (52.0%) of SCZ patients and *n* = 40 (80.0%) of controls had at least one filled tooth, respectively. In addition, *n* = 5 (10.0%) of SCZ patients and *n* = 2 (4.0%) of controls had severe tooth loss, respectively. Male subjects had significantly lower DMF-S (74.52 ± 39.72 vs. 90.67 ± 39.1; *p* = 0.020) and DMF-T (16.52 ± 8.12 vs. 20.52 ± 8.32; *p* = 0.031) scores compared to females, while only numerical tendencies were shown for D (6.92 ± 2.33 vs. 8.78 ± 2.10; *p* = 0.31), M (7.45 ± 6.57 vs. 9.19 ± 7.45; *p* = 0.43) and F (4.12 ± 2.30 vs. 3.24 ± 2.62; *p* = 0.54) scores and the number of crowns, respectively (2.13 ± 3.31 vs. 2.53 ± 1.10; *p* = 0.245) (Table 2).

In the context of female subjects, patients with SCZ had significantly higher D (9.19 ± 1.45 vs. 4.61 ± 0.89; *p* = 0.009) and significantly lower F (2.14 ± 0.67 vs. 5.09 ± 0.99; *p* = 0.16) scores, while only numerical differences were observed for DMF-S scores (90.66 ± 48.69 vs. 76.86 ± 37.24; *p* = 0.174), DMF-T scores (20.52 ± 1.82 vs. 17.33 ± 7.59; *p* = 0.129), M scores (9.19 ± 1.78 vs. 7.71 ± 1.72; *p* = 0.66) and the number of crowns (2.42 ± 1.18 vs. 2.76 ± 0.83; *p* = 0.145), respectively. For male subjects, patients with SCZ had significantly higher DMF-S (74.52 ± 7.38 vs. 50.62 ± 7.40; *p* = 0.023), D (7.44 ± 0.69 vs. 3.86 ± 0.81; *p* < 0.001), M (7.44 ± 1.38 vs. 4.10 ± 0.95; *p* = 0.045) and DMF-T (16.51 ± 1.51 vs. 12.31 ± 1.51; *p* = 0.045) scores but significantly lower F (1.62 ± 0.41 vs. 4.27 ± 0.66; *p* = 0.002) scores compared to the controls (Table 2.). Only numerical differences were shown for the number of crowns (1.27 ± 2.56 vs. 2.56 ± 5.10; *p* = 0.21).

In the context of smoking habits, DMF-S (64.10 ± 41.22 vs. 97.59 ± 29.25; *p* < 0.001), DMF-T (64.10 ± 41.22 vs. 97.59 ± 29.25; *p* = 0.006) and M (6.17 ± 6.28 vs. 9.48 ± 6.80; *p* = 0.017) scores were significantly higher in individuals who smoked >10 cigarettes/day. On the other hand, based on alcohol consumption (0–3 units/week vs. ≥4 units/week), coffee consumption (0–1 cups/day vs. ≥2 cups/day) and vitamin supplementation status (taking vs. not taking them), no significant differences were found for either of the dental status indices.

### 3.3. Periodontal Status Parameters

The summary of periodontal status findings corresponding to SCZ patients and controls is presented in Table 3. Patients with SCZ had significantly higher plaque indices (56.96 ± 23.19 vs. 27.44 ± 17.53; *p* < 0.001), BOP% (58.96 ± 22.89 vs. 23.56 ± 17.53; *p* < 0.001), PD (2.84 ± 0.67 vs. 2.19 ± 0.49; *p* = 0.024) and AL (3.39 ± 1.72 vs. 2.49 ± 0.76; *p* < 0.001) values compared to controls (Table 3). Similar findings were shown when comparing male (plaque indices: 53.86 ± 23.34 vs. 26.43 ± 15.25, *p* < 0.001; BOP%: 55.36 ± 22.08 vs. 23.61 ± 19.32, *p* < 0.001; PD: 3.02 ± 0.68 vs. 2.13 ± 0.53, *p* = 0.009; AL: 3.34 ± 1.39 vs. 2.35 ± 0.73, *p* = 0.032) and female SCZ patients and control subjects separately (plaque indices: 61.30 ± 22.91 vs. 28.85 ± 14.09, *p* < 0.001; BOP%: 64.00 ± 23.47 vs. 23.50 ± 15.85, *p* < 0.001; PD: 2.71 ± 0.65 vs. 2.29 ± 0.45, *p* = 0.038; AL: 3.34 ± 1.39 vs. 2.35 ± 0.73; *p* = 0.012)) (Table 3). Only numerical differences were shown for all periodontal status parameters when compared between male and female participants (plaque indices: *p* = 0.600; BOP%: *p* = 0.875; PD: *p* = 0.196; AL: *p* = 0.116).

In the context of smoking habits, plaque indices (36.33 ± 20.85 vs. 61.91 ± 25.36; *p* < 0.001), BOP% (35.37 ± 24.82 vs. 61.05 ± 24.74; *p* < 0.001), PD (2.37 ± 0.59 vs. 3.50 ± 0.14; *p* < 0.001) and AL (2.75 ± 1.11 vs. 3.59 ± 2.22; *p* < 0.001) were significantly higher in individuals who smoked >10 cigarettes/day. Subjects who consumed ≥4 units/week of alcohol also had significantly worse periodontal status parameters (plaque indices: 33.59 ± 20.79 vs. 51.17 ± 24.73, *p* < 0.001; BOP% (31.73 ± 23.51 vs. 51.19 ± 26.92; *p* < 0.001), PD (2.32 ± 0.59 vs. 2.71 ± 0.69; *p* = 0.005) and AL (2.68 ± 1.08 vs. 3.21 ± 0.23; *p* = 0.008)). In contrast, coffee consumption (0–1 cups/day vs. ≥2 cups/day) and vitamin supplementation status (taking vs. not taking them) had no significant effect on either of the periodontal status parameters.

## 4. Discussion

Mental disorders are a heterogeneous group of illnesses, which have multidimensional impacts on an individual’s health and well-being [36]. Psychiatric patients are considered a special-needs and vulnerable patient group, requiring tailored approaches from both general health and dental healthcare professionals [37]. SCZ patients are often hindered in the context of procuring oral healthcare; in addition, preventive dental services are often not a part of the care provided to institutionalized persons [38]. The systematic review and meta-analysis of Yang et al. described SCZ as an independent risk factor for poor dental health (i.e., higher D, M and DMF-T values but lower F values compared to the non-SCZ population) [8]. Patients in acute episodes or in relapse have irregular behaviors and activities, which limit them from paying attention to regular oral hygiene. Inversely, poor oral health may further aggravate the overall health status and QoL of SCZ patients [8,9]. If patients are capable of performing activities of daily living, attention to oral self-care (i.e., the use of fluoride-containing toothpaste) and professional preventive fluoridation strategies during the attendance of a dental visit, are crucial. However, due to the negative symptoms of SCZ, both self-cleaning practices and motivations to visit dental healthcare professionals are hindered considerably [39].

In our single-center observational study, the oral health status of fifty SCZ patients were comprehensively assessed and compared to age- and sex-matched healthy controls to offer valuable insights for tailored preventive and rehabilitative measures. The majority (~80%) of patients received antipsychotic medication in addition to mood stabilizers and/or sedato-hypnotic drugs. By reducing salivary flow rate, many pharmaceuticals, including antipsychotic medications, antidepressants and benzodiazepines, contribute to disadvantageous shifts in the oral microbiota and to the development of dental caries [40]. The dose–response relationship between antipsychotics and the deterioration of oral health has been described previously [41]. Furthermore, as many SCZ patients may have various mental and/or physical comorbidities—especially in individuals ≥50 years of age—many additional drugs (e.g., antihypertensives, parasympatholytics, antihistamines) may need to be taken, further exacerbating side effects through anticholinergic and anti-alpha-adrenergic receptor activities, such as hyposalivation and drowsiness [8,42]. On the other hand, maxillo-facial dystonia or tardive dyskinesia—a major side effect of antipsychotic drugs—may further limit orofacial functionality in SCZ patients [43]. It has also been described that taking anti-SCZ drugs may lead to changes in the oral microbiota composition, which promote pro-inflammatory processes in the mouth, leading to worse periodontal disease outcomes [44]. Furthermore, as a cumulative consequence of drug adverse effects, poor oral hygiene, overgrowth of oral *Candida* spp. and the increasing prevalence of dysphagia with age, SCZ patients are at a higher risk of developing hospital-acquired/aspiration pneumonia, where members of the oral microbiota are often seen as important etiological factors [45].

In line with our initial hypothesis, the main dental status parameters (i.e., DMF-S, DMF-T) corresponding to caries experience were significantly higher among SCZ patients compared to controls. A high prevalence of participants affected by missing and filled teeth were observed in both groups (84.0% and 72.0%, and 52.0% and 80.0%, respectively), which is consistent with previous epidemiological studies (~80%) in psychiatric patients [46,47]. Interestingly, male participants overall had better dental status, which is in contrast to the findings of many previous studies [8,9]. No significant differences were shown for the number of crowns between SCZ patients and controls; this number was used as an empirical measure of an individual’s control over their oral health versus having many decayed/filled teeth. Similarly, SCZ patients presented with significantly worse clinical periodontal status parameters (i.e., plaque %, BOP%, PD and AL) compared to their matched controls, concurrent with our initial hypothesis; on the other hand, statistical differentiation among the sexes was not observed in the context of periodontal health indicators. High M and DMF-T scores may be explained by the dental care strategies often utilized for SCZ patients; often, tooth extraction is chosen as treatment due to convenience and the difficulties associated with their care, foregoing more work-intensive, tooth-conserving methods, which would require the cooperation of the patients [48]. However, teeth loss is a major contributor to impaired mastication, oral functionality and QoL; furthermore, the relationship between severe tooth loss and cognitive decline has also been highlighted [49]. The oral rehabilitation of these patients through restorative protocols is considerably more cost-intensive compared to the timely application of preventive measures [50]. In addition, due to the limited illness perception and adherence to medical advice in SCZ patients—complicated by xerostomia and parafunctional habits (e.g., bruxism)—the success rates of restorative treatments may be limited. For example, the use of partial or total removable dentures may be impractical or even impossible [20].

This study also assessed the influence of several anamnestic parameters on oral health parameters; consequently, the deleterious effect of smoking >10 cigarettes/day was shown in our sample, leading to higher DMF-S, M and DMF-T values. Smokers also presented with significantly higher values in all measured periodontal status indicators. Tobacco use is a known contributor to xerostomia, and the detrimental effects of tobacco use on gingival health and on the progression of periodontal disease has also been described [51,52]. In addition, alcohol consumption of over 4 units/week also had a detrimental effect on periodontal health; as SCZ patients may often use drinking as a coping strategy, this could inadvertently further exacerbate oral health [53]. The study of Tezal et al. assessed the periodontal status (expressed in AL) in the context of alcohol consumption and found a dose–response relationship between AL levels and 5, 10, 15 and 20 drinks/week using the data of the Third National Health and Nutrition Examination Survey (NHANES III) in the US [54]. On the other hand, our initial hypotheses were not confirmed regarding vitamin supplement use and coffee consumption habits, as variance in those regards did not lead to statistically significant differences in oral health parameters. Similar to our findings, the Mendelian randomization study of Liao et al. also failed to show a strong association between coffee-consuming behavior and periodontitis, indicating a risk increase of ~1% [55]. The study of Saleh et al. established the role of regular vitamin and supplement consumption on periodontal health in adults, using the “BigMouth” dental data repository: among the 21 supplements surveyed, only the consumption of multivitamins and iron showed substantial benefits for periodontal health [56]. Furthermore, the role of vitamin D in the maintenance of periodontal health—both in the context of bone metabolism and as an anti-inflammatory agent—was described [57].

While there have been numerous publications reporting on the dental and periodontal health of SCZ patients worldwide, well-founded comparisons with our data is made difficult as studies from Central–Eastern Europe (and from similar healthcare settings) are scarce; this has been highlighted by the systematic review of Khokhar et al. (2016) [58] and the systematic review and meta-analysis of Kisely et al. (2018) [36]. A single-center, case-control study from Spain reported similar mean dental status parameters (D: 7.26, M: 9.10, F: 1.30, DMF-T: 17.74) and a correspondingly high prevalence of missing and filled teeth in SCZ patients, while no significant differences were noted on the basis of the patient’s sex [33]. A Greek observational study reported considerably higher mean DMF-T scores (23.35 ± 8.36), with a high burden of M teeth in SCZ patients, although a clear delineation was shown between outpatients and long-term inpatients. Additionally, the authors noted strong and significant correlations between the negative symptom subscale of the Brief Psychiatric Rating Scale (BPRS) values, DMF-T scores and simplified Oral Hygiene Index (OHI-15), respectively [59]. In a cross-sectional study from China, the relationship of dental status with cognitive and mental status was assessed in inpatient SCZ patients >50 years of age, with a battery of neuropsychological scales [60]. Mean DMF-T values were 12.99 ± 8.86 in their patient population (prevalence of caries and tooth loss: 83.1% and 83.3%), which were significantly higher in individuals who smoked or who used to smoke; DMF-T values and M values showed significant negative correlation with the Mini-Mental State Examination Scale (MMSE) score, but significant positive correlation with age and the Global Deterioration Scale (GDSRANK) scores, respectively [60]. A Japanese observational study assessed the oral health status (using the DMF-T index, calculus index [CI], debris index [DI] and Revised Oral Assessment Guide [ROAG]) of hospitalized SCZ patients; significant negative correlations were shown between DMF-T values (mean: 21.7 ± 7.3, higher in males), chlorpromazine equivalents (CPZE) and Barthel index (BI) (denoting mental illness severity), while positive correlations were found with age and length of hospitalization [61]. Another Spanish case-control study compared the dental and periodontal health status among SCZ patients and controls without psychiatric illnesses: mean dental status indices (D: 4.39, M: 5.66, F: 3.53, DMF-T: 13.51) were lower compared to our study, while the mean periodontal health (expressed using the community periodontal index [CPI]) score was 2.32. SCZ patients had worse outcomes both in the case of caries and in periodontal status compared to control participants. Their study also found significant correlations between DMF-T, CPI scores and the negative subscale of the Positive and Negative Syndrome Scale (PANSS) [62].

A comparative study from Taiwan involving SCZ patients in psychiatric long-term care institutions and individuals from the general public assessed the dental and periodontal status (using CPI) of the subjects [63]. The study has showed that SCZ patients had significantly higher DMF-T values (13.94 ± 8.48 vs. 8.39 ± 7.01), edentulism (5.0% vs. 1.7%) and CPI indices (CPI = 3 35.9% vs. 5.1%) and a lower number of remaining teeth (17.66 ± 8.83 vs. 23.23 ± 6.62) compared to the general population, while no significant differences were found based on the individual’s sex [63]. A recent population-based cohort study in Taiwan involved over 3600 individuals with newly diagnosed SCZ who developed periodontal disease within a year of their diagnosis. They showed a significant association between female sex (adjusted OR [aOR]: 2.24), receipt of first-generation (aOR: 1.89) and second-generation (aOR: 1.33) antipsychotics, blood pressure medications (aOR: 1.91), anticholinergics (aOR: 1.24) and the development of periodontal disease [64]. In addition to tooth loss, it was suggested that poor oral health may lead to cognitive impairment via another pathway: due to the progression of periodontal disease, a chronic and systemic inflammatory burden is present, predominantly due to the presence of *Porphyromonas gingivalis* virulence factors [65]. As a consequence, pro-inflammatory cytokines (IL-1, IL-6, TNF-α) are released, contributing to neuroinflammation [66].

Patients affected by SCZ should be considered as a priority group to receive dental healthcare services to improve and maintain their QoL, functionality, and societal integration. This requires targeted interventions as a part of health policy initiatives, in addition to the heightened awareness of dentists directly interacting with these patients. Furthermore, closer interprofessional collaboration is warranted between the providers of dental and psychiatric health services [67]. In the following, we provide a set of concise and practical recommendations to ensure the success of dental consultations and treatments for SCZ patients: (*i*) If a patient with a history of SCZ presents at the dental office, consultation with the psychiatrist managing the patient is recommended, with a special focus on reviewing medical history, medications and the current status of the patient. (*ii*) Dentists should be familiar with the possible drug–drug interactions between medicines commonly prescribed for SCZ patients and drugs used during dental treatments, as some drugs (e.g., lithium) may need to be temporarily suspended. (*iii*) An organized, consistent routine should be developed for present and future appointments, where individual steps are familiar for the patient. (*iv*) Initiatives should be put forth for a relaxing atmosphere in the treatment area by reducing stimulation of the patient (either by music, background noises or unnecessary contact) as much as possible. (*v*) The comfort level of the patients should continually be checked, with healthcare professionals being mindful of akathisia (restlessness of the extremities) and avoiding arguing with or antagonizing the patient. (*vi*) If cooperation with the patient becomes difficult, involvement of the psychiatrist is recommended. (*vii*) If possible, the presence of a family member and/or caregiver in the treatment area is recommended. (*viii*) The patient’s explanation of their oral hygiene practices should be surveyed and corrected as needed. (*ix*) Oral hygiene and self-care instructions should be explained in a way that is understandable at the patient’s awareness level. (*x*) The patient’s oral health status should be closely monitored, and in case of an exacerbation, interventions should be carried out rapidly to prevent further deterioration of periodontal status and tooth loss. (*xi*) Dentists should be aware of the characteristic findings to look for (e.g., complaints of xerostomia, ulcers, involuntary movements, dysphagia, candidiasis) during the assessment of SCZ patients. (*xii*) If the patient experiences severe orofacial dyskinesia, a bite block should be used. (*xiii*) Dental interventions should be controlled for possible bleeding. (*xiv*) Precautions should be taken to avoid postural hypotension, and equipment should be available to monitor vital signs. (*xv*) Special care should be taken to avoid the use of adrenaline-containing anesthetics (due to the risk of a hypertensive crisis) and atropine (due to the risk of severe anticholinergic effects) [8,9,20,67].

The results of our study should be interpreted in the context of its limitations: this study was performed with a relatively small sample size (which may include false-negative and false-positive findings). Additionally, our sample was taken from a pool of patients of a single clinical center, which limits the generalizability of the results. Our study may also be affected by selection bias, which is a characteristic of epidemiological studies performed in tertiary-care health facilities. Furthermore, some important socio-demographic (e.g., socio-economic status, living conditions, highest level of educational attainment, current of previous occupations, relationship status/availability of social support), medical (duration of illness, symptom severity, number of relapses, characteristics of disease management, level of adherence, other medical and/or psychiatric comorbidities, a complete panel of drugs taken [except for the drugs for which qualitative data were available], therapeutic drug levels) and lifestyle (workup of oral hygiene habits before the instructions, diet, risk-taking behaviors [except for the habits for which qualitative data were available]) information on the subjects—which could act as confounders in observational epidemiological studies—were not assessed.

## 5. Conclusions

Our present single-center case-control observational study included a comprehensive assessment of dental and periodontal status in SCZ patients vs. controls, which highlighted the overall poor oral health in individuals affected by the disease. The underlying effects of relevant clinico-epidemiological correlates were also assessed, highlighting smoking as a risk factor for worse dental health and alcohol consumption as a risk for worse dental and periodontal health, respectively. Furthermore, male patients had better dental status parameters, a finding dissimilar to many existing epidemiological reports. The results of the present clinical study underline that SCZ patients are at high risk of developing oral disorders, highlighting the need for improved access to oral healthcare services and targeted preventive interventions in Hungary.

## Figures and Tables

**Table 1:** Anamnestic data corresponding to patients with SCZ and controls.

	Patients with SCZ		Controls	
	*n*	%	*n*	%
Cigarettes/day				
0–5	11	22.0	37	74.0
6–10	20	40.0	9	18.0
11–20	15	30.0	3	6.0
≥20	4	8.0	1	2.0
Alcohol consumption (units/week)				
0	9	18.0	19	38.0
1–3	24	48.0	23	46.0
4–10	17	34.0	8	16.0
Coffee consumption (cups/day)				
0	3	6.0	13	26.0
1	16	32.0	22	44.0
2	20	40.0	9	18.0
≥3	11	22.0	6	12.0
Takes vitamin supplements				
No	40	80.0	20	40.0
Yes	10	20.0	30	60.0

**Table 2:** Dental status parameters of patients with SCZ and controls.

	Patients with SCZ					Controls				
	D	M	F	DMF-T	DMF-S	D	M	F	DMF-T	DMF-S
Total	8.18 ± 5.17	8.18 ± 7.73	1.84 ± 0.29	18.20 ± 8.36	81.30 ± 40.16	4.18 ± 4.22	5.62 ± 6.61	4.62 ± 3.98	14.42 ± 8.21	61.64 ± 40.56
Gender										
Male	7.44 ± 0.69	7.47 ± 1.38	1.62 ± 0.41	16.51 ± 1.51	74.52 ± 7.37	3.86 ± 0.81	4.10 ± 0.95	4.27 ± 0.66	12.31 ± 1.51	50.62 ± 7.40
Female	9.19 ± 1.45	9.19 ± 1.78	2.14 ± 0.67	20.52 ± 1.82	90.66 ± 8.69	4.61 ± 0.89	7.71 ± 1.72	5.09 ± 0.99	17.33 ± 7.59	76.88 ± 37.24

D: decayed; M: missing; F: filled; DMF-T: decayed, missing and filled teeth; DMF-S: decayed, missing and filled surfaces.

**Table 3:** Periodontal status parameters of patients with SCZ and controls.

	Patients with SCZ				Controls			
	Plaque Index (%)	BOP%	PD	AL	Plaque Index (%)	BOP%	PD	AL
Total	56.96 ± 23.19	58.96 ± 22.89	2.84 ± 0.67	3.39 ± 1.72	27.44 ± 17.53	23.56 ± 17.53	2.19 ± 0.49	2.49 ± 0.76
Gender								
Male	53.86 ± 23.34	55.36 ± 22.08	3.02 ± 0.68	3.34 ± 1.39	26.43 ± 15.25	23.61 ± 19.32	2.13 ± 0.53	2.35 ± 0.73
Female	61.30 ± 22.91	64.00 ± 23.47	2.71 ± 0.65	3.44 ± 2.15	28.85 ± 14.09	23.50 ± 15.85	2.29 ± 0.45	2.70 ± 0.78

BOP%: bleeding on probing, PD: pocket depth, AL: attachment loss.

## Data Availability

All data generated during the study are presented in this paper.
